# Supplementary material for: The Exon Junction Complex Core Represses Cancer-Specific Mature mRNA Re-splicing: A Potential Key Role in Terminating Splicing
Source: Int J Mol Sci. 2021 Jun 17;22(12):6519. doi: 10.3390/ijms22126519 (PMC8234774; doi:10.3390/ijms22126519)
Supplement: Supplementary file 1 [file ijms-22-06519-s001.zip › ijms-1195587-supplementary.pdf]

Communication

The Exon Junction Complex Core Represses Cancer-specific Mature mRNA Re-splicing: A Potential Key Role in Terminating Splicing

Yuta Otani [1,2], Ken-ichi Fujita [1], Toshiki Kameyama [1,3,*] and Akila Mayeda[1,*]

[1] Division of Gene Expression Mechanism, Institute for Comprehensive Medical Science, Fujita Health University, Toyoake, Aichi 470-1192, Japan
[2] Laboratories of Discovery Research, Nippon Shinyaku Co., Ltd., Kyoto, Kyoto 601-8550, Japan
[3] Present address: Department of Physiology, Fujita Health University, School of Medicine, Toyoake, Aichi 470-1192, Japan
* Correspondence: tkame@fujita-hu.ac.jp (T.K); mayeda@fujita-hu.ac.jp (A.M.)

Contents

Table S3
(Tables S1 and S2 are uploaded in separate Excel files)

Fig. S1

Fig. S2

Fig. S3

Fig. S4

Fig. S5

Table S3 List of the synthetic oligonucleotides used in the experiments (see also Table S2).

**siRNAs (sense sequences) for cellular knockdown** (T: deoxyribonucleotide)		
EIF4A3 siRNA #1	ID: s18876 (Thermo Fisher Scientific)	Knockdown (KD) of EIF4A3 (shared in Table S2)
EIF4A3 siRNA #2	ID: s18877 (Thermo Fisher Scientific)	
RBM8A (Y14) siRNA #1	ID: s532199 (Thermo Fisher Scientific)	KD of RBM8A
RBM8A (Y14) siRNA #2	ID: s532200 (Thermo Fisher Scientific)	
MAGOH siRNA #1	ID: s226590 (Thermo Fisher Scientific)	KD of MAGOH
MAGOH siRNA #2	ID: s8466 (Thermo Fisher Scientific)	
CASC3 (MNL51) siRNA #1	ID: s22392 (Thermo Fisher Scientific)	KD of CASC3
CASC3 (MNL51) siRNA #2	ID: s22393 (Thermo Fisher Scientific)	
UPF1 siRNA #1	ID: s11927 (Thermo Fisher Scientific)	KD of UPF1
UPF1 siRNA #2	ID: s11928 (Thermo Fisher Scientific)	
NXF1 siRNA #1	ID: s20533 (Thermo Fisher Scientific)	KD of UPF1
NXF1 siRNA #2	ID: s20532 (Thermo Fisher Scientific)	
ACINUS siRNA	5′-gaggccuucuggauugacaTT -3′ (Integrated DNA Technologies)	KD of ACINUS
PININ siRNA	5′-ggagcaagggcauuuacuaTT-3′ (Integrated DNA Technologies)	KD of PININ
RNPS1 siRNA	5′-caaaggcuaugcguacguaTT-3′ (Integrated DNA Technologies)	KD of RNPS1
SAP18 siRNA	5′-gaacugacaagcuuaguaaTT-3′ (Integrated DNA Technologies)	KD of SAP18
**Primer DNAs for plasmid construction** (F: forward primer, R: reverse primer)		
RBM8A coding-F	5’-ACAAGTGGCCTCTGAGGCCCCACCATGGCGGACGTGCTAGATCT-3’	Construction for final pSB-RBM8A
RBM8A coding-R	5’-TGACGGGCCTGACAGGCCTCAGCGACGTCTCCGGTCTG-3’	
**Primer DNAs for mRNA products assay** (F: forward primer, R: reverse primer)		
GAPDH-F	5’-GCACCGTCAAGGCTGAGAAC-3’	PCR for detection of GAPDH cDNA
GAPDH-R	5’-TGGTGAAGACGCCAGTCCA-3’	
EIF4A3-F	5’-GATCTTGGCTCCCACAAGAG-3’	PCR for detection of EIF4A3 cDNA
EIF4A3-R	5’-TAGTCACCGAGAGCAAGCAG-3’	
TSG101-F	5’-GAAGTTATCATTCCCACAGCTC-3’	PCR for detection of spliced TSG101
TSG101-R	5’-GACGTACATGCTTCAGGAAGAC-3’	
TSG101Δ154-1054-F	5’-TACAAATACAGAGACCTAACTCTCCC-3’	PCR for detection of re-spliced TSG101
TSG101Δ154-1054-R	5’-GACGTACATGCTTCAGGAAGAC-3’	
RBM8A-F	5’-ATTCATCTCAACCTCGACAGG-3’	PCR for detection of RBM8A cDNA
RBM8A-R	5’-CAAATCCTGGCCATTGAGTC-3’	
MAGOH-F	5’-CAAAGAGGATGATGCATTGTG-3’	PCR for detection of MAGOH cDNA
MAGOH-R	5’-TGTGTTCATCTCCAATGACGA-3’	
CASC3-F	5’-ACCACCGCCTCATCTGTATC-3’	PCR for detection of CASC3 cDNA
CASC3-R	5’-TGGGCGGGGTTATAGTAGGT-3’	
UPF1-F	5’-GCAACGGACGTGGAAATACT-3’	PCR for detection of UPF1 cDNA
UPF1-R	5’-CTTGTGCAGGGTCACCTCTT-3’	
GAS5-F	5’-CTTGCCTGGACCAGCTTAAT-3’	PCR for detection of GAS5 cDNA
GAS5-R	5’-CAAGCCGACTCTCCATAC-3’	
NXF1-F	5’-GATTCTTTGCGAGCCTTCACC-3’	PCR for detection of NXF1 cDNA
NXF1-R	5’-AGGAGGAGATGACAGACGACAACC-3’	
ACINUS-F	5’-GACGATCATCTAGGGTCAGACAG-3’	PCR for detection of ACINUS cDNA
ACINUS-R	5’-GGAAGGTGTTTCTTGATCCTCTT-3’	

Left, middle, and right columns: names, sequences, and purpose of the oligonucleotides, respectively.

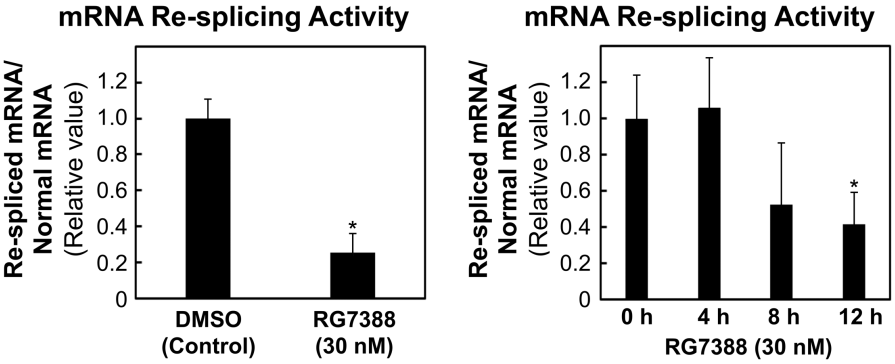

Figure S1. Activation of TP53 by the MDM2 inhibitor RG7388 induces the repression of TSG101 mRNA re-splicing activity: To evaluate the re-splicing activity with TSG101 mRNA, the extracted total RNAs from MCF-7 cells were analyzed by RT–qPCR using specific primer sets (see Figure 1a). The RG7388 concentrations and culture time after RG7388 addition were indicated. The histograms represent the means ± standard deviations of three replicates (*$p < 0.05$).

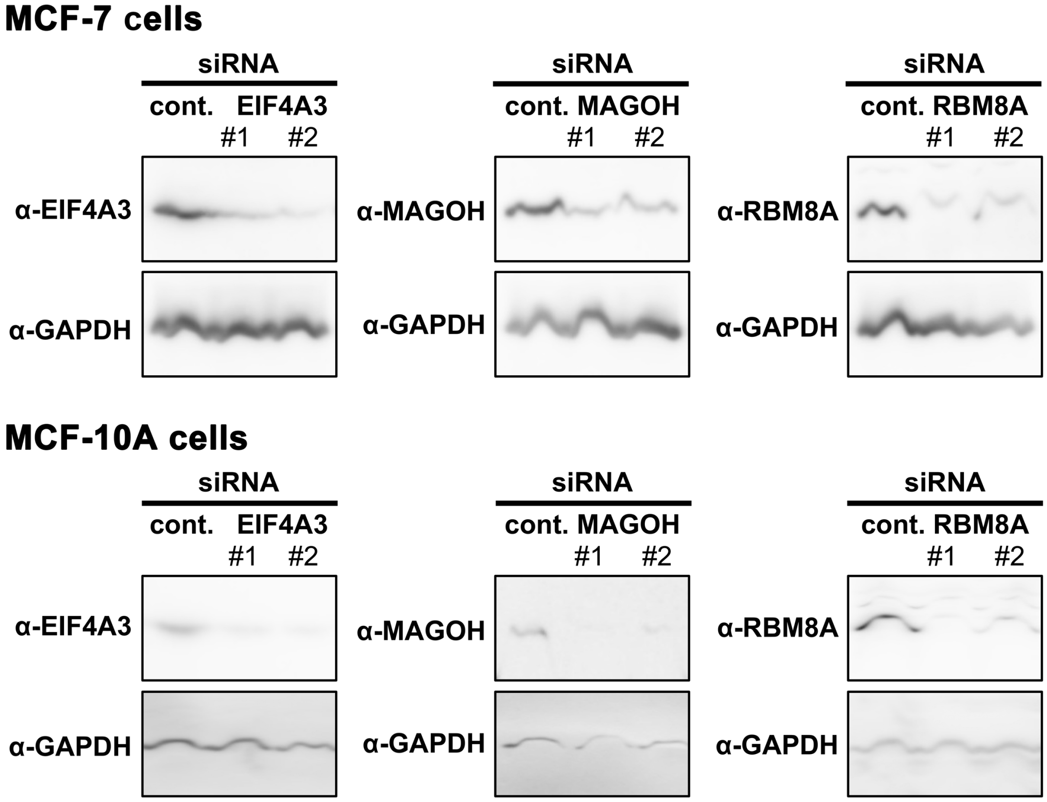

Figure S2. The endogenous EJC-core proteins are effectively depleted by the siRNA-mediated knock-down: MCF-7 and MCF-10A cells were transfected with siRNA targeting EIF4A3, RBM8A, MAGOH or control siRNA, and whole cell extracts were analyzed by immunoblotting with the indicated specific antibodies.

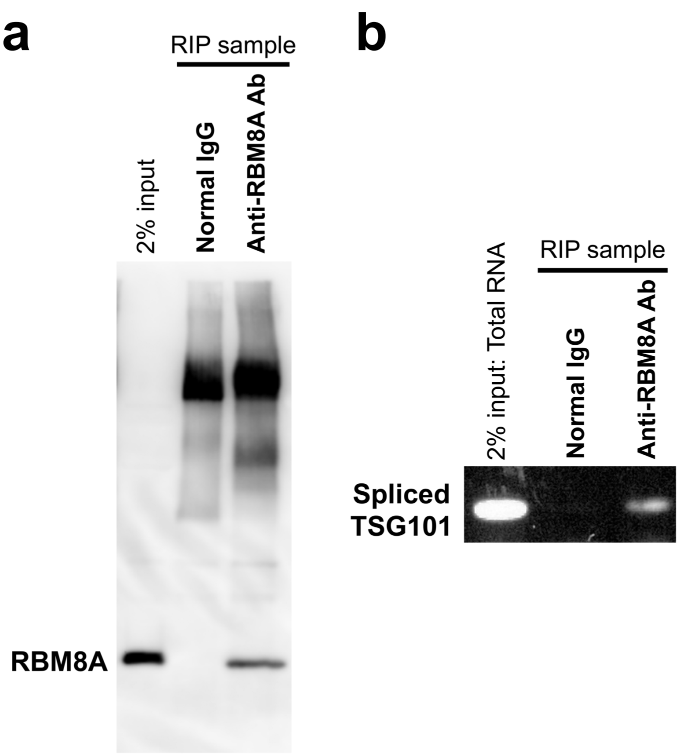

Figure S3. The EJC core factor RBM8A binds TSG101 mRNAs in MCF-7 cells: (**a**) RBM8A-bound mRNAs were separated from cell lysates of MCF-7 cells by RNA immunoprecipitation (RIP). Normal rabbit IgG was used as a negative control of the immunoprecipitation. Two independent RIP assays were performed and a representative immunoblot is shown here. (**b**) The RIP samples were analyzed by RT–PCR using a primer set targeting spliced TSG101 mRNA. The PCR products were visualized by agarose gel electrophoresis.

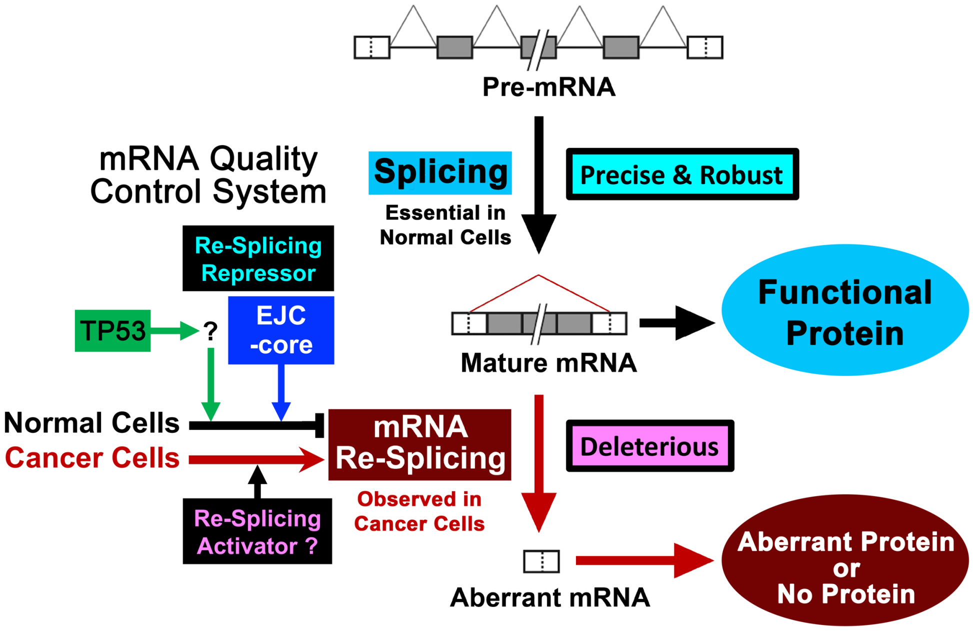

Figure S4. Model of the regulation system of cancer-specific mRNA re-splicing: TP53 represses mRNA re-splicing [10,15; see reference list in the main text], however, the downstream repressor has not been identified. Our discovery of EJC-core as a repressor of mRNA re-splicing implies a mechanism for terminating pre-mRNA splicing in normal cells, which is essential to maintain quality of mRNA.

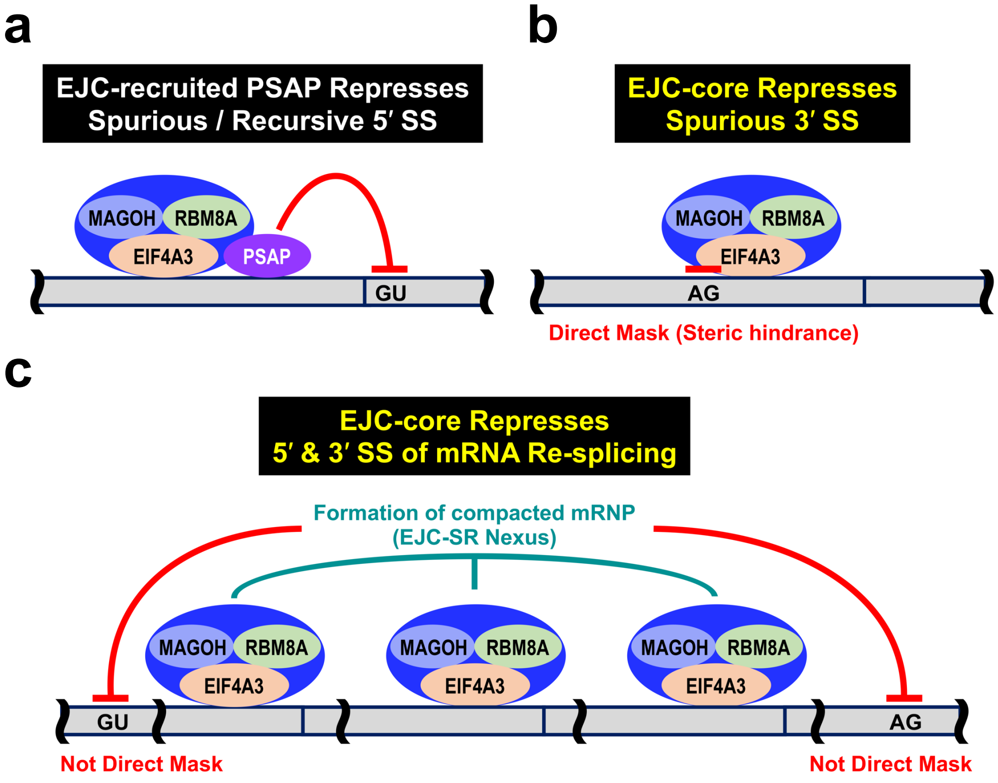

Figure S5. Similarity and distinction with other findings of the EJC-induced splicing repression: Recently, it has been reported that EJC deposition on mRNA represses nearby spurious splice sites [22; see reference list in the main text] and downstream recursive 5′ splice site [23]. (**a**) EJC-recruited PSAP complex (PNN, SAP18 and RNPS1) represses spurious 5′ splice site (SS) [22] and downstream recursive 5′ SS (but the requirement of PSAP is dependent on the substrate of recursive splicing) [23]. (**b**) On the other hand, nearby spurious 3′ SS is repressed by direct binding of the EJC-core, which causes steric hindrance [22]. (**c**) Here we showed that EJC-core represses exonic 5′ SS and 3′ SS used in mRNA re-splicing. Previous CLIP-Seq data suggest that the repression of mRNA re-splicing is not due to the direct binding of EJC-core on the 5′ SS and 3′ SS [24]. We propose that EJC-core deposition triggers the formation of compacted mRNP leading to the prevention of further re-splicing on mRNA (see Figure 6).
